# Vitamin K Deficiency Embryopathy from Hyperemesis Gravidarum

**DOI:** 10.1155/2015/324173

**Published:** 2015-08-12

**Authors:** Andrew S. Lane, Jennifer L. Stallworth, Kacey Y. Eichelberger, Kenneth F. Trofatter

**Affiliations:** ^1^Department of Obstetrics and Gynecology, University of South Carolina School of Medicine Greenville, Greenville Health System, 890 W. Faris Road, Suite 470, Greenville, SC 29605, USA; ^2^Greenwood Genetic Center, 14 Edgewood Drive, Greenville, SC 29605, USA; ^3^Division of Maternal Fetal Medicine, Department of Obstetrics and Gynecology, University of South Carolina School of Medicine Greenville, Greenville Health System, 890 W. Faris Road, Suite 470, Greenville, SC 29605, USA

## Abstract

A 21-year-old primigravida had a pregnancy complicated by hyperemesis gravidarum (HG) beginning at 7-week gestation. Despite medical therapy, she lost 18% of her prepregnancy weight. Early ultrasound at 14 weeks demonstrated a flattened facial profile with nasal hypoplasia (Binder phenotype) consistent with vitamin K deficiency from HG. She had a percutaneous endoscopic gastrojejunostomy tube placed for enteral feeding at 15-week gestation. At repeated anatomy ultrasound at 21-week gestation, delivery, and postnatal pediatric genetics exam, nasal hypoplasia was consistent with vitamin K deficiency embryopathy from HG. Nausea and vomiting of pregnancy is a common condition. HG, the most severe form, has many maternal and fetal effects. Evaluation of vitamin K status could potentially prevent this rare and disfiguring embryopathy.

## 1. Introduction

Nausea and vomiting of pregnancy (NVP) is a common condition affecting 70–85% of pregnancies [1]. Hyperemesis gravidarum (HG) is a severe form of NVP most commonly defined as persistent vomiting not related to other causes, ketonuria, and weight loss greater than 5% of prepregnancy weight [2]. HG affects 0.5–2% of pregnancies and is the most common indication for hospitalization in the first trimester of pregnancy [3]. We present the earliest diagnosed case of vitamin K deficiency embryopathy due to HG. Our objective is to raise awareness of a rare fetal complication of HG.

## 2. Case Presentation

A 21-year-old African American primigravida experienced nausea and vomiting of pregnancy (NVP) since 7-week gestation. She was diagnosed with hyperemesis gravidarum (HG) based on NVP, ketosis, and loss of greater than 5% of prepregnancy weight. Her HG was resistant to intravenous fluids, ondansetron, promethazine, and metoclopramide. She was referred to our maternal-fetal medicine department at 14-week gestation.

**Figure 1:**
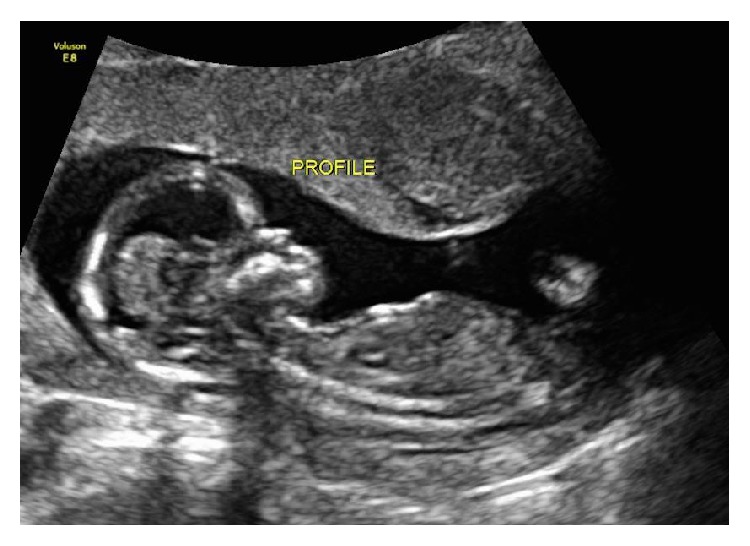
Profile at 14-week gestation shows prominent forehead and nasal hypoplasia.

At our initial assessment, other diagnoses associated with nausea and vomiting in pregnancy were excluded: gastroenteritis, hepatitis, appendicitis, pancreatitis, pyelonephritis, and diabetic ketoacidosis. Serum amylase, lipase, liver transaminases, creatinine, and electrolytes were all within normal limits. Urinalysis showed large ketones. At this time, her weight was at its nadir with a loss of 17 kg from her prepregnancy weight (18%). She did not have any other medical comorbidities associated with vitamin K deficiency in pregnancy such as inflammatory bowel disease, celiac disease, bulimia, bariatric surgery, or chronic pancreatitis. She was not taking any warfarin derivatives, vitamin A, or vitamin E. Early anatomy assessment at 14 weeks demonstrated a prominent forehead and flattened facial profile (Figure 1). There was no evidence of bleeding dyscrasias.

At 15-week gestation, she was admitted to the hospital due to worsening HG. An oncologic surgeon placed a percutaneous endoscopic gastrojejunostomy (PEG-J) tube for enteral feeding. She was started on an enteral feeding supplement containing vitamin K. During the admission, hypokalemia was corrected and she received daily injections of multivitamin, thiamine, and folate with her intravenous fluids.

**Figure 2:**
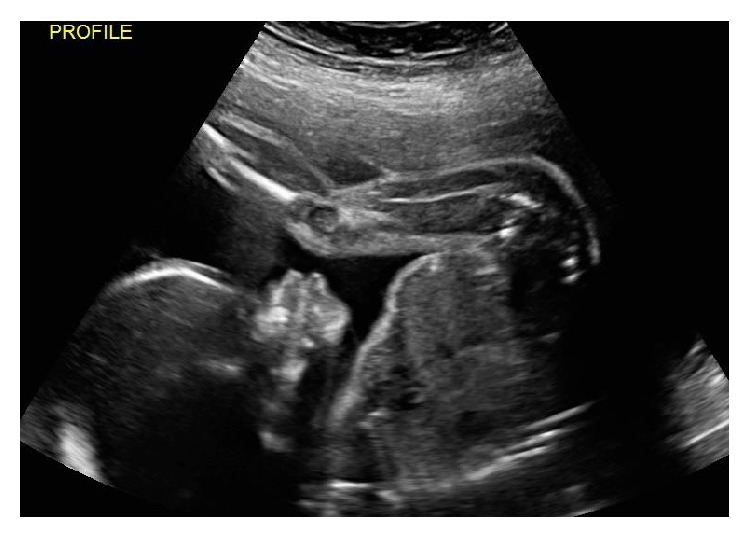
Anatomy assessment at 21-week gestation shows continued flat facial profile with nasal hypoplasia.

**Figure 3:**
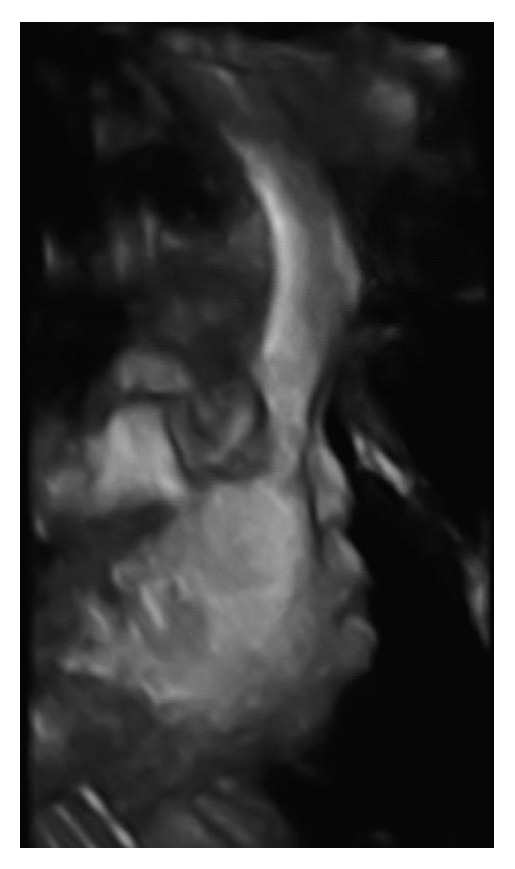
3D ultrasound at 21 weeks shows striking nasal hypoplasia.

Anatomy ultrasound at 21 weeks again demonstrated nasal hypoplasia, flat facial profile, and prominent forehead (Figures 2 and 3). No other anomalies were identified. The patient declined genetic amniocentesis but consented to cell-free fetal DNA testing, which showed an XY male with low risk for trisomy 21, trisomy 18, and trisomy 13. The combination of continued nasal hypoplasia and maternal history made the diagnosis of vitamin K deficiency embryopathy. The patient's vitamin K level at that time was 245 pg/ml (reference 80–1160 pg/mL), suggesting replenishment of the patient's vitamin K stores from enteral feeding.

Examination at delivery showed a hypoplastic nose, high arched palate without cleft, and flattened facial features consistent with ultrasound findings. Pediatric developmental occupational therapy found no feeding deficits. The neonate passed his newborn hearing screening and was discharged home with his mother on day 3 of life.

**Figure 4:**
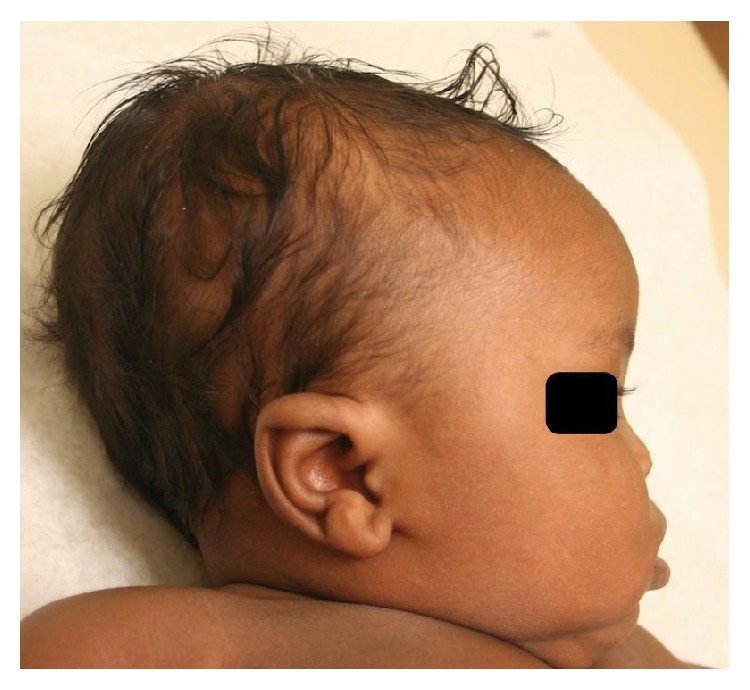
Facial profile 2 months after delivery shows nasal hypoplasia consistent with previous ultrasounds.

A postnatal examination by a pediatric geneticist confirmed the diagnosis of vitamin K deficiency embryopathy (Figure 4). Bone scans of the hands, lower legs, hips, and skull did not show chondrodysplasia punctata. Long-term prognosis of the infant is promising.

## 3. Discussion

Vitamin K is a cofactor in the conversion of vitamin K dependent proteins, which play many important roles in coagulation (prothrombin, factors: VII, IX, and X, and proteins: C, S, and Z) [4]. Coagulation abnormalities caused by vitamin K deficiency can be detected by a prolonged prothrombin time (PT) and international normalized ratio (INR) due to predominate effect on factor VII. Proteins induced in vitamin K absence (PIVKA-II) is a more sensitive marker as it will be elevated prior to any changes in global coagulation assays such as PT and is preferred over direct measurement of vitamin K [5]. PT is a convenient screening test, which can be followed by PIVKA-II to confirm deficiency.

Nasal hypoplasia is known as Binder phenotype. Binder phenotype is often found with chondrodysplasia punctata (CDP), a radiographic finding comprised of stippled epiphyses (punctate calcifications on radiography). An infant with solely nasal hypoplasia is given the diagnosis of Binder phenotype and one with nasal hypoplasia and stippled epiphyses is given the diagnosis of Binder phenotype with CDP. The pathogenesis of Binder phenotype is either genetic or nongenetic. Nongenetic causes are related to vitamin K deficiency through mechanisms such as HG, warfarin use, or malabsorption. Genetic causes are myriad. Some genetic causes include rhizomelic chondrodysplasia punctata 1, Zellweger syndrome, and pseudowarfarin embryopathy [4]. Due to the patient's clinical history, family pedigree, physical exam, and imaging, the geneticist confirmed the diagnosis of Binder phenotype and did not recommend any further genetic testing.

We performed a MEDLINE search of English speaking journals from the time of the first reported case in 1997 to March 2015, using the following search terms: “binder phenotype,” “vitamin K deficiency in pregnancy,” “vitamin K deficiency embryopathy,” and “vitamin K deficiency in hyperemesis gravidarum.” Our search returned with 3 articles with a total of 8 cases [4, 6, 7]. This is the 9th published case of Binder phenotype related to HG, the first detected at such an early gestation, and the first with accompanying ultrasound images. Review of the prior 8 cases showed that, much like our case, most newborns suffered only from the cosmetic effects of the embryopathy [4, 7].

ACOG recommends correction of ketosis and vitamin deficiency in HG patients requiring IV hydration [2]. Providing adequate nutrition when a pregnant patient is unable to maintain oral intake can be daunting. Nasogastric tubes are associated with high patient dissatisfaction and high failure rate and PICC lines are associated with infectious morbidity [8]. PEG-J tubes allow for a method of enteral feeding that can easily be concealed that is better-tolerated on the long term [9, 10].

In summary, we recommend that vitamin K deficiency should be considered in any patient requiring hospitalization for HG, as correction of a deficiency may prevent vitamin K deficiency embryopathy. Screening with PT and, if prolonged, confirmation with PIVKA-II seem a reasonable approach. Early fetal anatomy scans should include detailed facial anatomy to assess nasal hypoplasia. Due to the rarity of these cases, the ideal replacement strategy is unknown. Therefore, a nutritionist should be part of the treatment team to ensure proper repletion of vitamin K. Daily replacement with vitamin K 10 mg subcutaneously until correction of laboratory values has been used successfully in case reports [11]. If enteral or parenteral feeding is required, it must include enough vitamin K to meet the daily AI of 90 mcg [5].
